# Rapid detection of low‐level HeLa cell contamination in cell culture using nested PCR

**DOI:** 10.1111/jcmm.13923

**Published:** 2018-10-24

**Authors:** Jun Lin, Lin Chen, Wenqian Jiang, Huilian Zhang, Yang Shi, Weiwen Cai

**Affiliations:** ^1^ Institute of Apply Genomics Fuzhou University Fuzhou China; ^2^ School of Basic Medical Sciences Fujian Medical University Fuzhou China; ^3^ College of Biological Science and Engineering Fuzhou University Fuzhou China

**Keywords:** cell cross‐contamination, HeLa, nested PCR

## Abstract

HeLa cells are a commonly used cell line in many biological research areas. They are not picky for culture medium and proliferate rapidly. HeLa cells are a notorious source of cell cross‐contamination and have been found to be able to contaminate a wide range of cell lines in cell culture. In this study, we reported a simple and efficient method for detecting the presence of HeLa cell contamination in cell culture. HPV‐18 was used as a biomarker. The cell culture supernatant was used directly as the template for nested PCR without extracting nucleic acid. By PCR amplification of the cell culture supernatant with the designed primers, we were able to detect the presence of HeLa cells in the culture. The sensitivity of this method can reach 1%, which is 10‐fold higher than Short tandem repeat sequence (STR) profiling. This simple, rapid, and “noninvasive” quality checking method should find applications in routine cell culture practice.

## 1 INTRODUCTION

HeLa cells are a cell line with unlimited proliferative capacity. It originated from cervical cancer tissue of an American woman in 1952.^1^, ^2^ As the first human cervical cancer cell line that could be cultured in vitro, HeLa cells have been widely used in cervical cancer research and played an important role in the research of cervical cancer cell biology and diagnosis, as well as treatment of cervical cancer.^3^ In addition, HeLa cells are a common model in cell biology and have contributed to numerous important discoveries such as the discovery of telomere's protective mechanism in chromosomes.^4^

When a cell line (called A) is contaminated by another cell line (called B), if B cells grow faster or have greater cellular activity, B will outgrow and eventually displace A after several generations.^5^ Unlike other cell lines, one of the characteristics of HeLa cells is their abnormally rapid proliferation rate. Hela cells can adapt to different growth conditions and different cell culture media, such as DMEM,^6^, ^7^ MEM,^8^ RMPI1640,^9^, ^10^ DMEM/F12K,^11^, ^12^ and are very easy to culture. Therefore, HeLa cells are one of the most important sources of cell cross‐contamination. From 1969 to 2004, 220 publications in the PubMed database were found to use improper HeLa‐contaminated cell lines.^13^ According to the latest statistics from the International Cell Line Authentication Committee (ICLAC), 488 cell lines have been found to be contaminated, of which 116 cell lines were contaminated and in some cases completely displaced by HeLa cells, accounting for 24% of the total number of known contaminated cell lines (Table S1). Therefore, in order to ensure the reliability of the experimental results, more and more scientific journals require the authors to submit a proof of cell purity before paper submission.^14^

There are many methods to detect cross‐contamination of cell lines, including isoenzymes zymogram analysis,^15^ human leucocyte antigen typing (HLA typing),^16^, ^17^ DNA fingerprinting,^18^ and short tandem repeat sequence profiling (STRs).^17^ Isoenzymes, commonly found in cells of higher organisms, are a group of enzymes that have the same catalytic activities, but differ in composition, physicochemical properties, and structure. Cells from different origins have different isozyme distributions. Analysis of gel electrophoresis banding patterns and relative migration distances for the individual isoforms of intracellular enzymes can be used to detect cross‐contamination of cells in cell banks.^19^, ^20^, ^21^, ^22^ However, studies have shown that the proportion of contaminated cells needs to have at least 10% of the total cell mass in order for the isoenzymes to be reliably differentiated.^20^ Human leucocyte antigen (HLA) complex is a major histocompatibility complex (MHC) in humans. There are quite a few differences in bases among HLA genes in different individuals, resulting in different numbers of restriction endonucleases recognition sites. After amplification of the target gene fragment by PCR, various restriction enzymes can be used to digest the amplified product to generate different digested products, and then the electrophoresis pattern is used for identification. It is also possible to carry out the analysis by hybridizing a probe to the amplification product.^23^, ^24^ Recently, the major HLA typing resolution is achieved by the Sequence‐Based Typing (SBT) method through direct DNA sequencing.^24^ For DNA fingerprinting, the variable numbers of tandem repeats (VNTRs) were amplified first to obtain the DNA profiles. Image analysis was then performed to determine the size of each amplicon of a locus on the agarose gel. Finally, the DNA profiles of all the samples were compared among each other to determine the difference.^25^ DNA fingerprinting is commonly used in the identification of human stem cell lines.^26^, ^27^ In recent years, STR profiling has been suggested as a golden method for authenticating human cell lines.^5^, ^28^, ^29^, ^30^, ^31^ STRs are tandemly repeated short DNA sequences, which are highly polymorphic in the human genome. The repeat sequence is usually 2‐6 bp in length.^32^ In the analysis of STR, the genomic DNA of target cell samples is extracted first, and then fluorophore‐labelled primers are used for PCR. The target STR sites can be amplified and the amplicons are marked with different colours of fluorescence. The amplified products were then separated by capillary electrophoresis to generate a multicolour fluorescence‐time profile based on differences in fluorescence colour and fragment size. After comparing the obtained STR profile with the reference in the database, it is possible to detect the existence of cell cross‐contamination.^17^, ^33^ Through the STR profiling, cross‐contamination of human cell lines can be identified.^5^ However, STR profiling method requires expansive equipment, the process is relatively complex, the cost more expensive, and the sensitivity low. Furthermore, STR is unable to detect cell contamination when the portion of contaminating cells was less than 10% of the total cell mixtures.^17^

Human papilloma virus (HPV) is a group of small, nonenveloped and double‐stranded DNA viruses belonging to the papillomaviridae family.^34^ HPV infections can lead to diseases such as cervical, anogenital, and head and neck cancer.^35^ In 1997, Walboomers^36^ confirmed that almost all cervical malignancies demonstrated oncogenic strains have the HPV gene, and HPV infection is considered to be an important cause of cervical cancer.^37^ In clinical testing for HPV, DNA or mRNA is usually extracted from the cervical specimen first. After amplification by PCR with specific primers, the presence of the HPV sequence can be identified by a probe to determine whether the sample is infected with HPV.^38^ There are approximately 200 HPV genotypes.^34^ Boshart^39^ detected the presence of the HPV‐18 gene in HeLa cell lines in 1984. As HPV‐18 positive human cervical cancer cell lines, there are two HPV‐18 DNA genomes in HeLa cells, with 7.8 kb and 6.9 kb in length. The latter has a 900 bp deletion.^39^

The HPV genome has a circular double‐stranded DNA structure, which including nonstructural proteins (E1, E2, E4, E5, E6, and E7), structural proteins (L1, L2), and a transcriptional control region.^40^ E1 and E2 participate in the initiation of viral DNA replication. E6 and E7 modulate the cell cycle control and contribute to viral genome maintenance. Both L1 and L2 are capsid proteins.^41^, ^42^, ^43^, ^44^ The human papillomavirus virions first penetrate the damaged area of the epithelium and infect the basal cells.^45^ Following viral entry and uncoating, HPV genomic DNA is maintained at a low‐copy number in the nuclei of basal cells.^46^ After leaving the basal membrane, HPV begins to replicate with the differentiation of the infected cells.^47^ Following the amplification of the genome, the synthesis of the capsid protein is triggered and the assembly of the virus is completed. Eventually, the progenitor virions are released externally with peeled keratinocytes.^40^ In HeLa cells, partial copy of the HPV‐18 genome is integrated at chromosome 8q24.^1^, ^48^ The integrated HPV contains L1, E1, E2, E6, and E7. We speculate that it may be possible to detect the genome of the released virus from the culture supernatant of HeLa cell. But there was no report of this before.

Most human cell lines do not contain the human papillomavirus virus. Therefore, we envisaged that it may be possible to detect the existence of HeLa cells by detecting the HPV‐18 gene sequence to confirm whether the cell line of interest is contaminated by HeLa cells. HPV is an obvious marker for HeLa cells. However, up to now, no one has ever used the HPV in culture medium as the biomarker to identify Hela cells. Therefore, in this study, PCR was performed directly on cell culture medium to amplify HPV‐18 sequence fragments to detect the presence of Hela cells. The method seems simple, but there are still some uncertainties. On the one hand, we chose to use the culture supernatants directly as the template for HPV‐18 sequence amplification without extraction of cell DNA, which can be regarded as a “noninvasive” type of detection. The other issue is the problem of detection sensitivity and time efficiency. The sensitivity of STR profiling can reach to 10%, but the STR profiling is time consuming with high cost. Whether this method can detect a tiny amount of HeLa cell contamination in a shorter period of time is another aspect of this study that needs to be explored. Based on the above considerations, we attempted to use a nested PCR assay to amplify the target DNA fragment. This method has high sensitivity and specificity, and it is suitable for the amplification of trace contaminants.^49^

## 2 MATERIALS AND METHODS

### 2.1 Cell cultures

The human cell lines HepG2, AGS, A549, HCT‐116, HGC‐27, NCI‐N87 were purchased from the cell bank of the Chinese Academy of Sciences. SNU‐216 was purchased from Nanjing CoBioer Technology Co., Ltd., China. Hela was purchased from National Institutes for Food and Drug Control of China. The base medium for cell line AGS and A549 cells is Kaighn's Modification of Ham's F‐12 Medium (F‐12K; GIBCO, REF.21127‐022, USA), SNU‐216 is RPMI‐1640 Medium (Shanghai BasalMedia Technologies Co., Ltd., REF.L220KJ, China), HCT‐116 is McCoy's 5A Medium (Shanghai BasalMedia Technologies Co., Ltd., REF.L220KJ, China), HGC‐27 and N87 RPMI‐1640 Medium (GIBCO, REF.A10491‐01, USA), Hela and HepG2 is Eagle's Minimum Essential Medium (Shanghai Basal Media Technologies Co., Ltd., REF.L220KJ, China). All these cells were cultured in their own base medium supplemented with 10% FBS (Biocell, REF.BC‐16‐1B, CHINA), penicillin (100 units/mL), streptomycin (100 μg/mL) and Mycoplasma inhibitor) (0.1 μg/mL, MP Biomedicals, USA) at 37°C in a humidified 5% CO_2_ atmosphere.

### 2.2 Doped cell culture

In this experiment, 24‐well cell culture plates were used. The density of plated cell was 1 × 10^5^ cells/well, with the proportion of Hela cells being 0%, 0.01%, 0.1%, 1%, 10%, 50%, and 100%. The cell supernatants were collected at 24 hours, 48 hours, and 72 hours later from culture at 37°C in a humidified 5% CO_2_ atmosphere without touching the adherence cells. We used cell supernatant directly as a template for subsequent PCR experiments, eliminating the steps of nucleic acid extraction. Cell culture supernatants can also be temporarily stored at −20°C until processed.

### 2.3 Nested PCR validation

**Figure 1:**
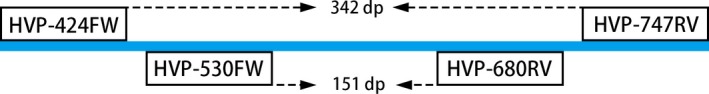
Primer positions for nested PCR

For the nested PCR assay, four primers HVP‐424FW, HVP‐747RV, HVP‐530FW, and HVP‐680RV (Table 1) were designed based on the HPV‐18 sequences available on Genbank database (accession number: NC_001357) to amplify an external fragment (324 bp) and an internal fragment (151 bp) of the HPV‐18 genome as shown in Figure 1. Positive controls consisted of pure HeLa cell culture supernatant, and sterile ultrapure water was used as the negative control.

**Table 1:** Primers for nested PCR assay

Primer name	Sequence
HVP‐424FW	5′ GGTGCCAGAAACCGTTGAATC 3′
HVP‐747RV	5′ CGTCGGGCTGGTAAATGTTGA 3′
HVP‐530FW	5′ CAACCGAGCACGACAGGAA 3′
HVP‐680RV	5′ ATTGCTCGTGACATAGAAGG 3′

For the HPV‐18 detection, two rounds of PCR assays were performed. The first round PCR mix solution was prepared to a final volume of 25 μL, containing 5 μL cell mixed culture supernatant, 1 μ mol L^−1^ forward primer, 1 μ mol L^−1^ reverse primer, and 12.5 μL 2 × Taq master Mix (CW Biotech, CW0682, China). Amplification reactions were initially incubated at 94°C for 5 minutes, followed by 35 cycles of 94°C/30 seconds, 54°C/30 seconds, and 72°C/20 seconds with a final extension at 72°C for 2 minutes. The second round PCR mix solution was prepared to a final volume of 25 μL, containing 0.5 μL of the first round PCR product, 1 μ mol L^−1^ forward primer, 1 μ mol L^−1^ reverse primer, and 12.5 μL 2 × Taq master Mix (CW Biotech, CW0682, China). Amplification reactions were initially incubated at 94°C for 5 minutes, followed by 35 cycles of 94°C/30 seconds, 50°C/30 seconds, and 72°C/15 seconds with a final extension at 72°C for 2 minutes. The PCR products were analysed by electrophoresis on a 2.5% agarose gel containing ethidium bromide and were visualized under UV light.

## 3 RESULTS

### 3.1 PCR results analysis

We used three types of cells: SNU‐216, HCT‐116, HGC‐27 mixed with HeLa cells. HeLa cells accounted for (cell number ratio) 0%, 0.01%, 0.1%, 1%, 10%, 50%, and 100%. After 24, 48, and 72 hours respectively, the supernatants were collected for PCR assay.

**Figure 2:**
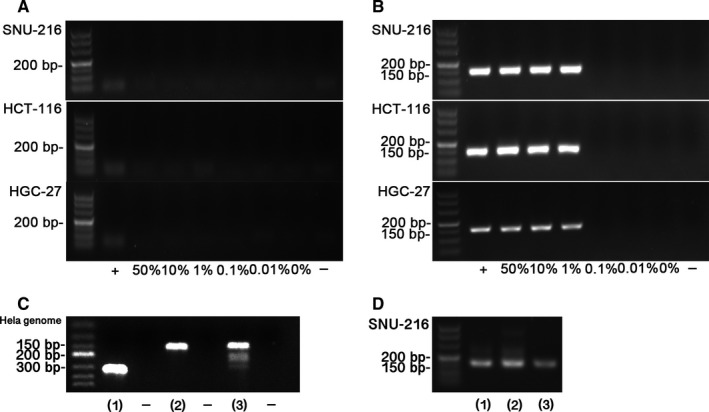
PCR assay results after 24 h. Nested PCR assay results for SNU‐216 cells, HGC‐27 cells, HCT‐116 cells doped with Hela and cultured for 24 h. A, The first round PCR results. After 24 h, mixed culture supernatants were collected for PCR assays for the proportion of HeLa at 0%, 0.01%, 0.1%, 1%, 10%, 50%, and 100% (+). B, The second round PCR results. After 24 h, mixed culture supernatants were collected for PCR assays for the proportion of HeLa at 0%, 0.01%, 0.1%, 1%, 10%, 50%, and 100% (+). C, Nested PCR assay using the Hela gemonic DNA. (1) HeLa gemonic DNA was detected by HVP‐424FW, HVP‐747RV (first round primers), (2) HeLa gemonic DNA was detected by HVP‐530FW HVP‐680RV (second round primers), (3) The nested PCR results of the Hela gemonic DNA. D, Detection of mixed culture supernatant with the proportion of HeLa cell at 1% in a SNU‐216 cell culture using three different PCR regents. (1) 2 × Taq master Mix (CW Biotech, CW0682, China), (2) Kodaq 2 × PCR MasterMix (Abm, G497‐dye, Canada), (3) Premix Taq (Takara, R004Q, Japan)

After cells were mixed and cultured for 24 hours, the mixed cell culture supernatants were collected for two round PCR. As illustrated in Figure 2, after the cells were mixed and cultured for 24 hours, the size of amplification product is about 150 bp that can be detected in the mixed culture supernatants when the proportion of HeLa was 1%, 10%, and 50%. The size of the amplified product was consistent with the target band of 151 bp (Figure 2C), indicating that the HPV‐18 sequence was successfully amplified. Without any optimization of amplification conditions, we were able to detect this target fragment by nested PCR using PCR kits from commercial suppliers (Figure 2D).

**Figure 3:**
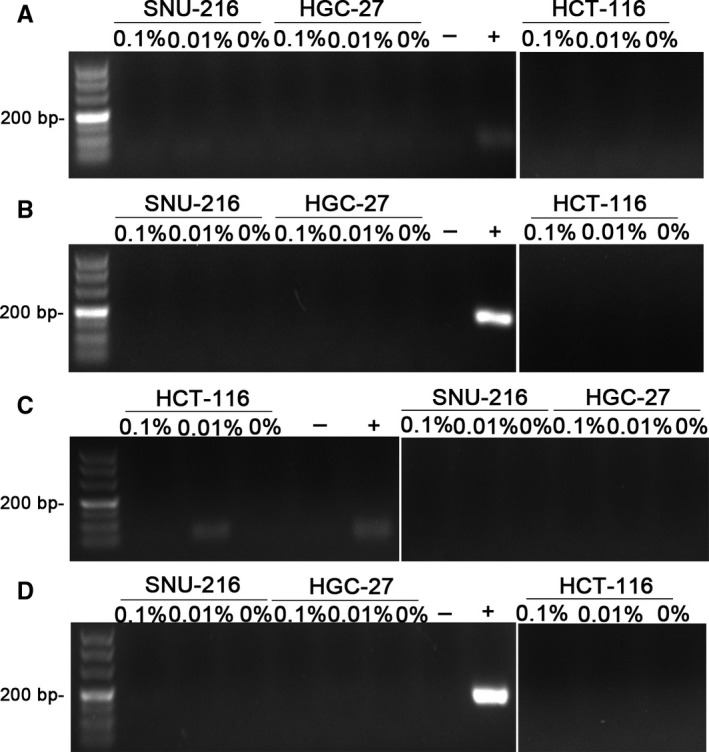
PCR assay results after 48 h and 72 h. Nested PCR assay results when SNU‐216 cells, HGC‐27 cells, HCT‐116 cells and Hela cells were mixed and cultured for 48 h and 72 h. A, The first round PCR results. After 48 h, mixed culture supernatants were collected for PCR assay when the proportion of HeLa was 0%, 0.01%, and 0.1%. B, The second round PCR results. After 48 h, mixed culture supernatants were collected for PCR assay when the proportion of HeLa was 0%, 0.01%, and 0.1%. C, The first round PCR results. After 72 h, mixed culture supernatants were collected for PCR assay when the proportion of HeLa was 0%, 0.01%, and 0.1%. D, The second round PCR results. After 72 h, mixed culture supernatants were collected for PCR assay when the proportion of HeLa was 0%, 0.01%, and 0.1%

We could not detect HeLa at low doping rate of 0%, 0.01%, and 0.1% after incubation of 48 hours (Figure 3). Even after 72 hours of incubation, no amplification products could be detected in the mixed cell culture supernatants when the proportion of HeLa was 0%, 0.01%, and 0.1%.

**Figure 4:**
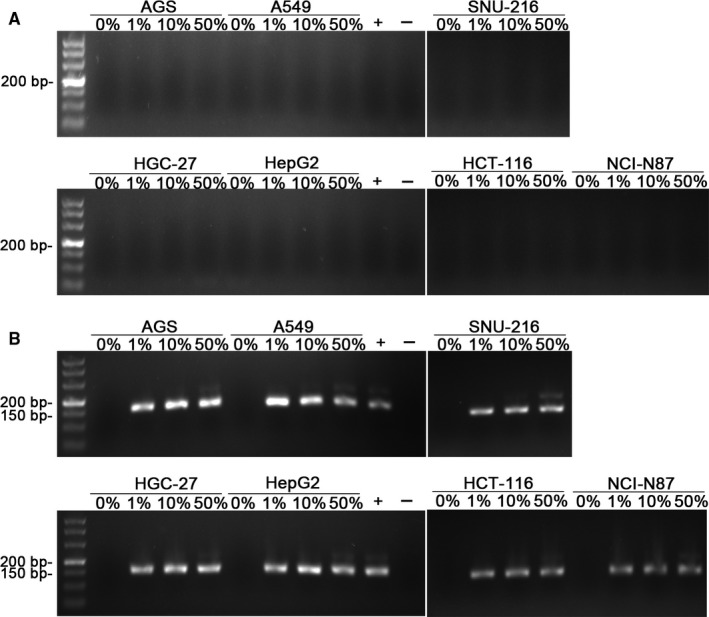
PCR assay results of seven types of cells after 24 h. Nested PCR assay results when seven kinds of cells were mixed with HeLa cells and cultured for 24 h. A, The first round PCR results. After 24 h, mixed culture supernatants were collected for PCR assay when the proportion of HeLa was 0%, 1%, 10%, 50%, and 100%. B, The second round PCR results. After 24 h, mixed culture supernatants were collected for PCR assay when the proportion of HeLa was 0%, 1%, 10%, 50%, and 100%

From these results, we estimated that the limit of detection sensitivity was between 0.1% and 1%. We then used seven cell lines: HepG2, AGS, A549, SNU‐216, HCT‐116, HGC‐27, N87 mixed with Hela cells to repeat the assay. The proportion of HeLa was 0%, 1%, 10%, 50%, and 100%. After 24 hours incubation, the mixed cell supernatants were collected for PCR assay. The results of the experiment are shown in Figure 4. These experiments indicated that as long as the number of Hela cells reached to 1%, the 150 bp amplification product can be detected in the mixed culture supernatant after 24 hours of incubation.

### 3.2 Sanger sequencing validation

**Figure 5:**
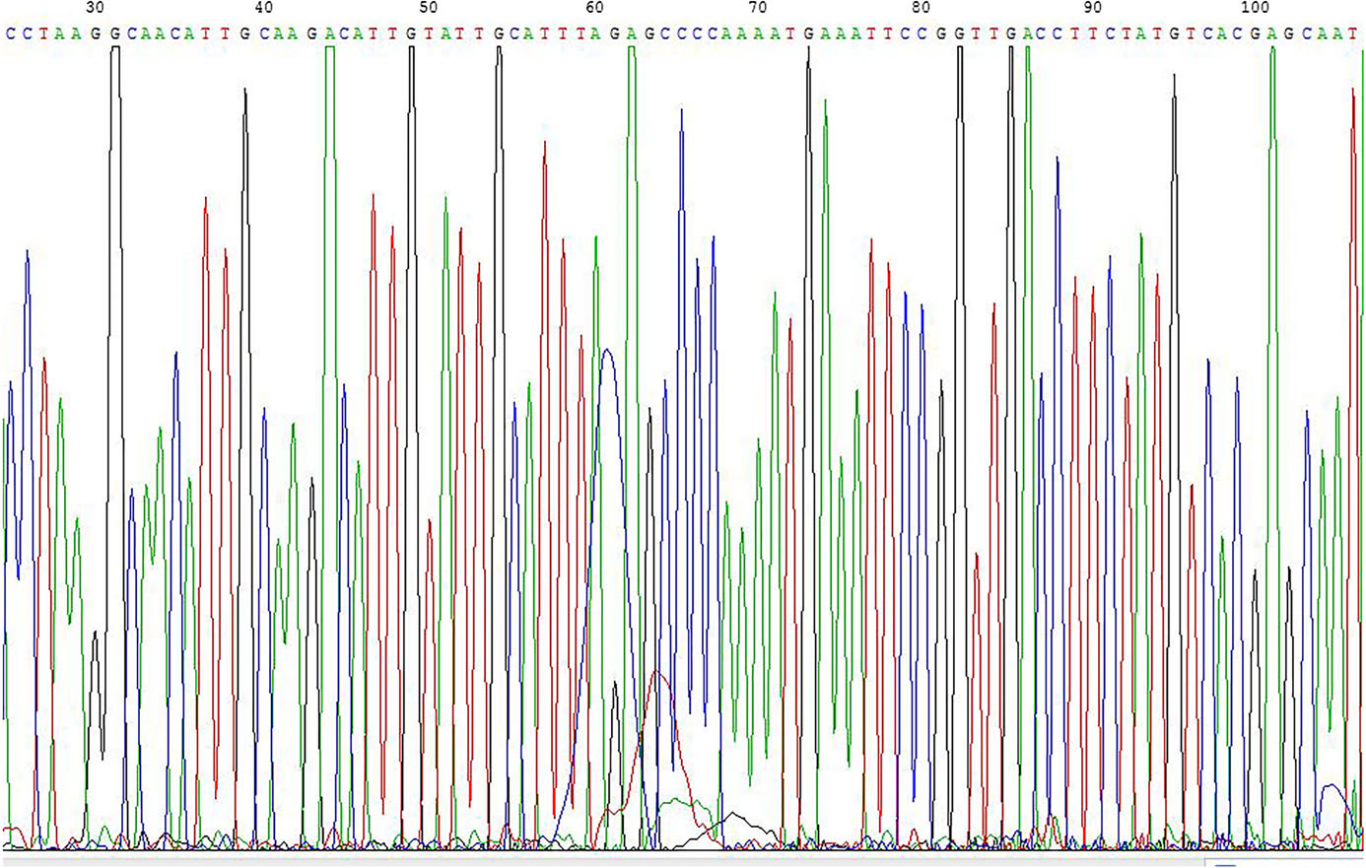
Sanger sequencing peak map of the HPV‐18 fragment

We used Sanger sequencing to verify the PCR amplicons. We selected some of the PCR products from mixed cell supernatant at 1% doping rate for sequencing validation. The sequencing results were confirmed using NCBI blast.^50^ The alignment showed that the sequencing results perfectly matched the sequence of 530‐680 bp of HPV‐18, which agreed with the expected PCR‐amplified fragment. The section of the Sanger sequencing peak map is shown in Figure 5.

### 3.3 Microscopic examination results

**Figure 6:**
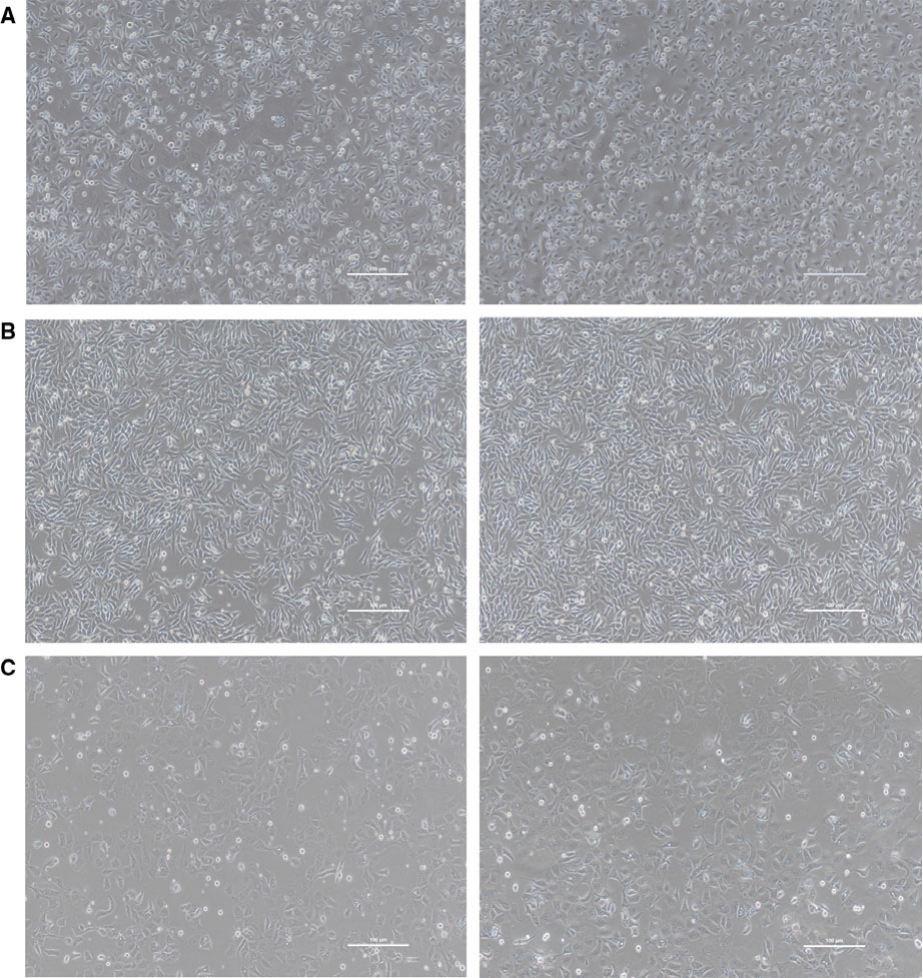
Microscopic examination results. A, AGS cells (left) and 99% of AGS cells are mixed with 1% of HeLa cells (right). B, HGC‐27 cells (left) and 99% of HGC‐27 cells are mixed with 1% of HeLa cells (right). C, SNU‐216 cells (left) and 99% of SNU‐216 cells are mixed with 1% of HeLa cells (right)

During the experiment, we randomly photographed Hela cells mixed with other cells at a 1% doping rate. By observing the morphology of the cells, we could not recognize that the cells had been contaminated with Hela cells (Figure 6). With the increase of cell passage times, Hela cells gradually outgrew the originally cells. If the morphology of the two cell type is close, the original cells may be completely displaced without notice and thus could lead to erroneous results.

### 3.4 STR profiling compared with PCR method

**Figure 7:**
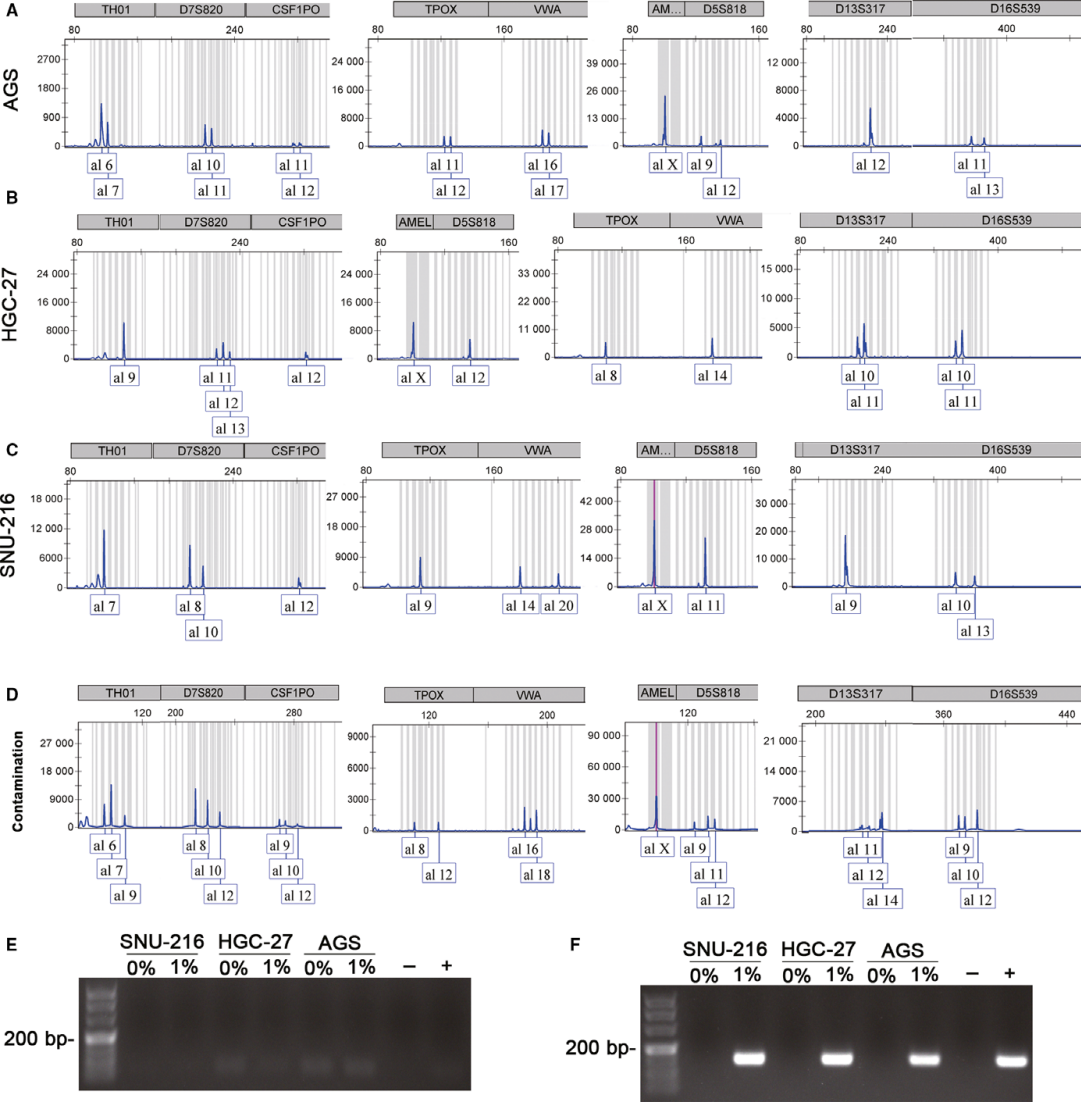
Short tandem repeat sequence (STR) profiling results and PCR results of cell culture supernatant after 24 h. STR profiling results. A, STR profiling results of mixed sediment of 99% AGS and 1% HeLa cells. B, STR profiling results of mixed sediment of 99% HGC‐27 and 1% HeLa cells. C, STR profiling results of mixed sediment of 99% SNU‐216 and 1% HeLa cells. Nested PCR assay results when SNU‐216 cells, HGC‐27 cells, AGS cells and Hela cells were mixed and cultured for 24 h. D, An STR profile of contamination cells. E, The first round PCR results of cell culture supernatant that cells were cultured after 24 h. HeLa cells were mixed and cultured with SNU‐216, HGC‐27, and AGS cells at a ratio of 0% and 1%. F, The second round PCR results of cell culture supernatant that cells were cocultured after 24 h. HeLa cells were mixed and cultured with SNU‐216, HGC‐27 and AGS cells at a ratio of 0% and 1%

In order to compare the sensitivity and convenience of STR profiling with the nested PCR method used in this study in detecting HeLa cell contamination, we mixed and cultured Hela cells with AGS cells, HGC‐27 cells, and SNU‐216 cells at a ratio of 1% and analysed adherent cells by STR profiling. The results are shown in Figure 7. At this low level of doping, Hela cell contamination was not detectable by STR profiling.

We then amplified the cell culture supernatant directly by nested PCR. The result (Figure 7) showed that amplification product around 150 bp could be stably detected in the supernatant when the proportion of HeLa was 1% after the cells were mixed and cultured for 24 hours.

Clearly, we found that, regardless of the growth rate of cells doped with HeLa cells, the HPV18 sequences could be amplified in the supernatant after 24 hours as long as the proportion of HeLa cells above 1%, whereas STR profiling was not able to detect the presence of HeLa cell contamination in the sample.

**Figure 8:**
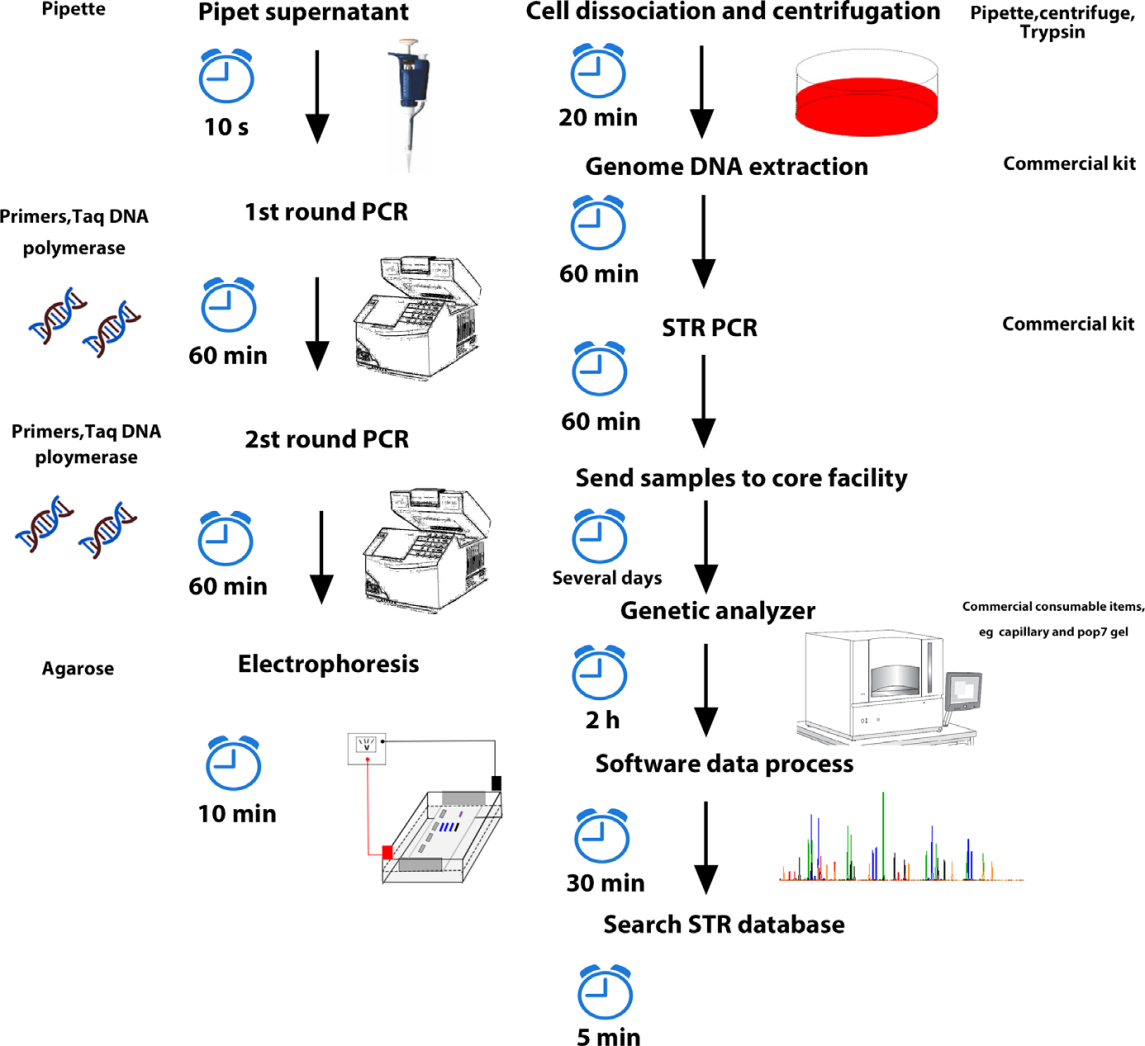
Workflow of nested PCR and STR for cell authentication

## 4 DISCUSSION

The problem of cross‐contamination in cell culture has plagued researchers for a long time. Many published results were proved unreliable because of the use of contaminated cell lines. Only until recently, scientists have begun to face up to this problem and develop various methods for detecting and identifying cell line cross‐contamination.

Because of its phenomenal growth rate, HeLa cells are one of the well‐known sources of cellular contaminations. As the first successfully cultivated human cancer cell lines, HeLa cells have been widely in various researches and are cultured in almost all basic research laboratories. Therefore, scientists should not only frequently observe the morphology and status of cells during cell culture, but also need to actively check by molecular methods whether cells being cultured in the laboratory are contaminated by HeLa cells if morphology differentiation is not a convenient option.

For the identification of HeLa cell contamination in this study, cell culture supernatant was used as a template for PCR. We used cell culture supernatant directly to amplify the HPV‐18 gene by nested PCR to detect the existence of HeLa cells in the culture. We found that when the cells were mixed and cultured for 24 hours or longer, the HPV‐18 sequence could be amplified in mixed cell culture supernatants when the proportion of HeLa was or higher than 1%. However, even up to 72 hours, there are no amplification fragments in mixed cell culture supernatants when the proportion of HeLa was 0.01% or 0.1%. We speculated that the cause might be the number of HeLa cells is too small when the proportion of HeLa was 0.1% or lower. At this level of contamination, there are about 10‐100 Hela cells in the mixed cell culture. This may not pose a serious concern in some experiments.

Compared with STR profiling, the PCR detection method adopted in this study had a much better sensitivity. Also, the process of STR profiling is cumbersome which requires the extraction of cell genome, quantification, amplification, and then fragment analysis. Moreover, it requires professional personnel to carry out experimental operations and data analysis, while PCR method only needs to amplify the supernatant of cell culture and then analysed by agarose gel electrophoresis (Figure 8). The experimental process of PCR assay is simple and easy to operate. Although quantitative PCR (qPCR) also has high sensitivity, it is limited by expensive instruments and reagents. Many laboratories do not have the ability to implement this equipment. The consequences of cell cross‐contamination are serious, which could lead to fouled experimental results. The PCR detection method demonstrated in this article can help scientists detect the existence of HeLa cells in the culture quickly. This simple method can be readily adopted by almost any biological laboratories and can be applied as a routine quality control tool.

Traditional methods of cell cross‐contamination detection involve the extraction of cellular genomic material. In order to extract cell genomic DNA, we need to digest cells and collect the cell pellet by centrifugation. In the process of cell harvest, the digestion time and resuspension are all possible variables that can cause cell damage. Also, a certain number of cells need to be killed in order to extract genomic DNA, which may not be practical for precious or slow‐growing cells. PCR assay of cell culture medium neatly obviates such need. Compared with traditional assays such as STR profiling, this method is a “noninvasive” type of detection that does not cause any damage to or loss of cells.

We can envisage that the fast, convenient, inexpensive, and easy to implement method we demonstrated in this study will allow scientists to simply collect culture supernatant for PCR amplification at any time during the process of culturing or passaging, and virtually in real‐time monitoring cell culture contamination. This simple approach may be applied to other cell culture system in which a specific marker for contaminating cells is available.

## CONFLICT OF INTEREST

The authors declare that they have no conflict of interest.
